# Supplementary material for: Neural representations of visual memory in inferotemporal cortex reveal a generalizable framework for translating between spikes and field potentials
Source: bioRxiv. 2026 Jan 4:2026.01.03.697516. Preprint. [Version 1] doi: 10.64898/2026.01.03.697516 (PMC12776368; doi:10.64898/2026.01.03.697516)
Supplement: 1 [file NIHPP2026.01.03.697516V1-supplement-1.pdf]

bioRxiv preprint doi: https://doi.org/10.64898/2026.01.03.697516; this version posted January 4, 2026. The copyright holder for this preprint (which was not certified by peer review) is the author/funder, who has granted bioRxiv a license to display the preprint in perpetuity. It is made available under aCC-BY 4.0 International license.

Supplementary Information

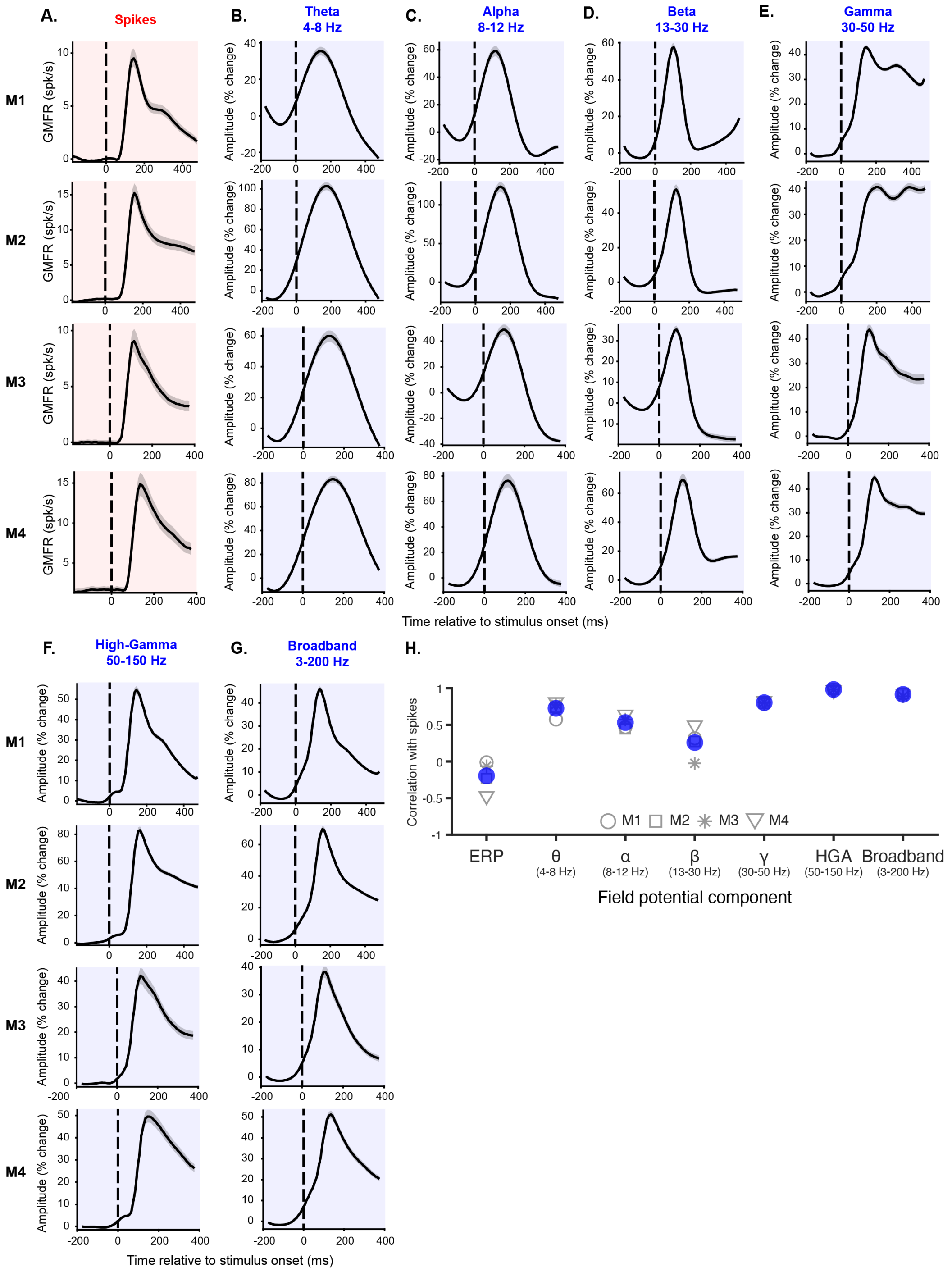

Figure S1. HGA captures population spiking vigor better than other features of the LFP. Each row is the neural data from one monkey. Spikes are shaded in red and features of the LFP in blue. Stimulus onset is marked with the dashed vertical black line. All measures are baseline subtracted with respect to activity in the window [-200, 0] and shown as a function of time relative to stimulus onset. Error shadows are bootstrapped 95% confidence intervals (see Methods). **A.** Grand mean firing rate. **B.** Amplitude of activity in the theta band (4-8 Hz). **C.** Amplitude of activity in the alpha band (8-12 Hz). **D.** Amplitude of activity in the beta band (13-30 Hz). **E.** Amplitude of activity in the gamma band (30-50 Hz). **F.** Amplitude of activity in the high-gamma band (50-150 Hz). **G.** Amplitude of broadband activity (3-200 Hz). **H.** Correlation between event-related time course of activity for each component of the LFP compared to spiking activity. Each shape is an individual monkey and the blue dots are the mean across the four monkeys.

bioRxiv preprint doi: https://doi.org/10.64898/2026.01.03.697516; this version posted January 4, 2026. The copyright holder for this preprint (which was not certified by peer review) is the author/funder, who has granted bioRxiv a license to display the preprint in perpetuity. It is made available under aCC-BY 4.0 International license.

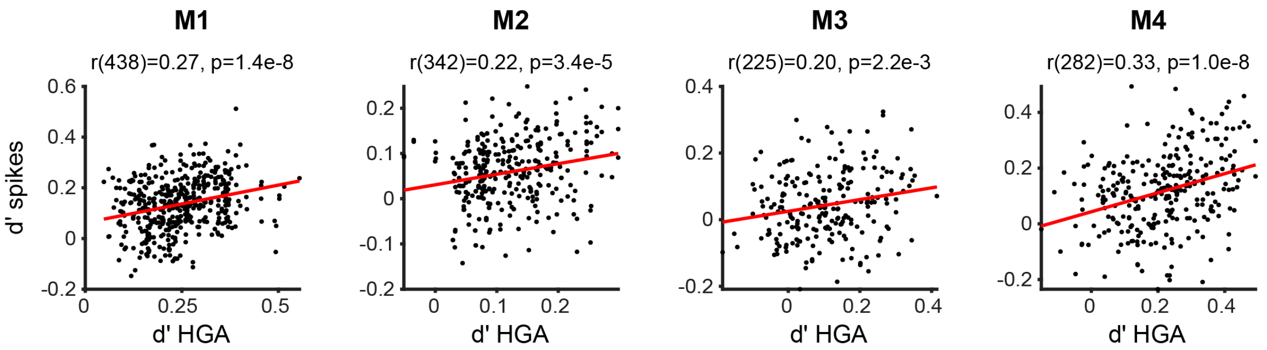

Figure S2. Spikes and HGA recorded on the same channel are weakly positively correlated. Pearson correlation between the d prime over images of a given unit and its corresponding channel's HGA for each monkey.

bioRxiv preprint doi: https://doi.org/10.64898/2026.01.03.697516; this version posted January 4, 2026. The copyright holder for this preprint (which was not certified by peer review) is the author/funder, who has granted bioRxiv a license to display the preprint in perpetuity. It is made available under aCC-BY 4.0 International license.

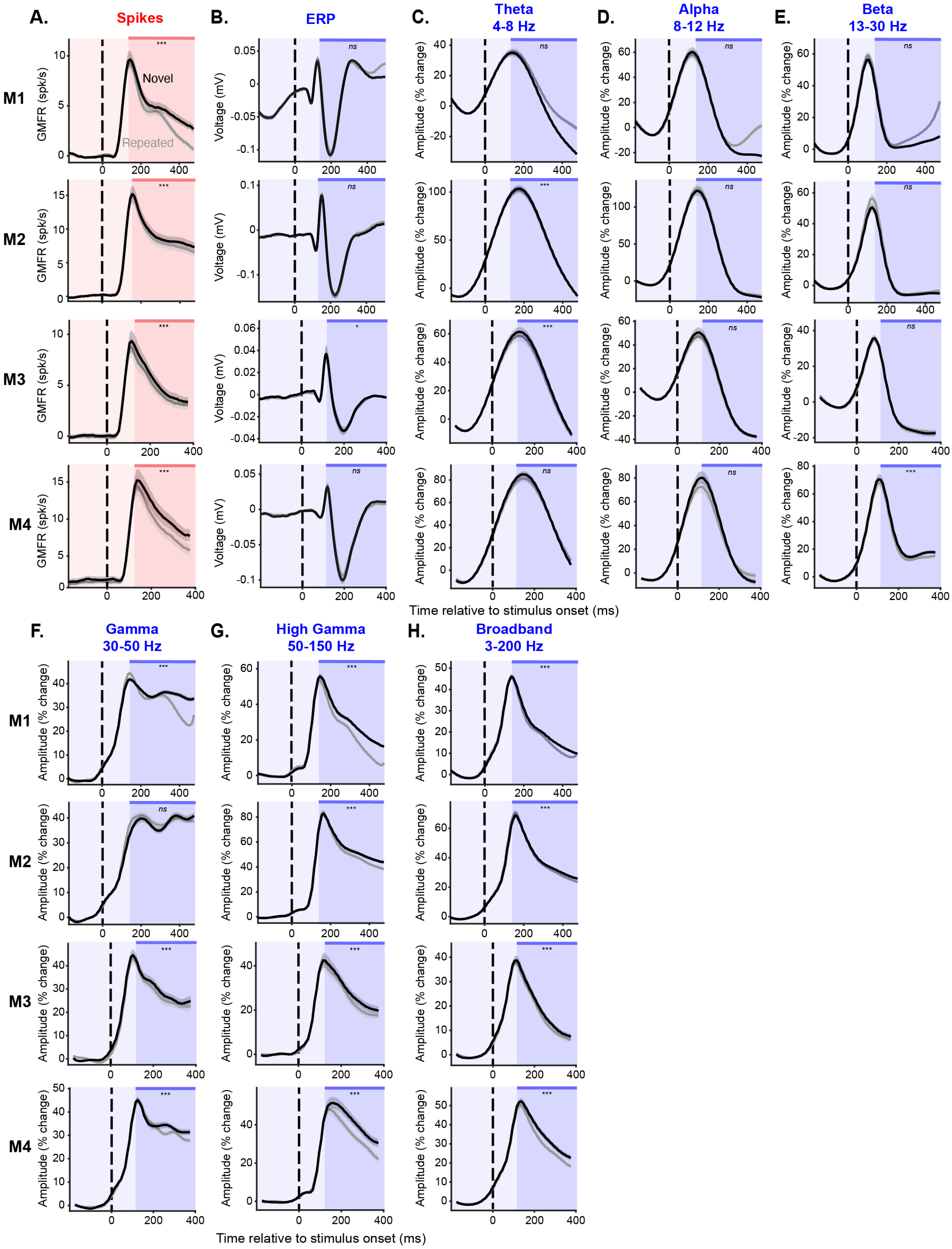

Figure S3. HGA captures repetition suppression better and more consistently than any other features of the LFP. Each row is the neural data from one monkey. Stimulus onset is marked with the dashed vertical black line. All measures are baseline subtracted with respect to activity in the window [-200, 0] and shown as a function of time relative to stimulus onset. Error shadows are bootstrapped 95% confidence intervals (see Methods). Significance tests are for a one-tailed Wilcoxon signed rank test. *ns*: non-significant, *: p<0.01, **: p<0.001, ***: p<0.0001. Shaded regions show the window used for significance testing (150 ms to the end of stimulus presentation). **A.** Grand mean firing rate. **B.** Magnitude of voltage fluctuation (ERP). **C.** Amplitude of activity in the theta band (4-8 Hz). **D.** Amplitude of activity in the alpha band (8-12 Hz). **E.** Amplitude of activity in the beta band (13-30 Hz). **F.** Amplitude of activity in the gamma band (30-50 Hz). **G.** Amplitude of activity in the high-gamma band (50-150 Hz). **H.** Amplitude of broadband activity (3-200 Hz).

bioRxiv preprint doi: https://doi.org/10.64898/2026.01.03.697516; this version posted January 4, 2026. The copyright holder for this preprint (which was not certified by peer review) is the author/funder, who has granted bioRxiv a license to display the preprint in perpetuity. It is made available under aCC-BY 4.0 International license.

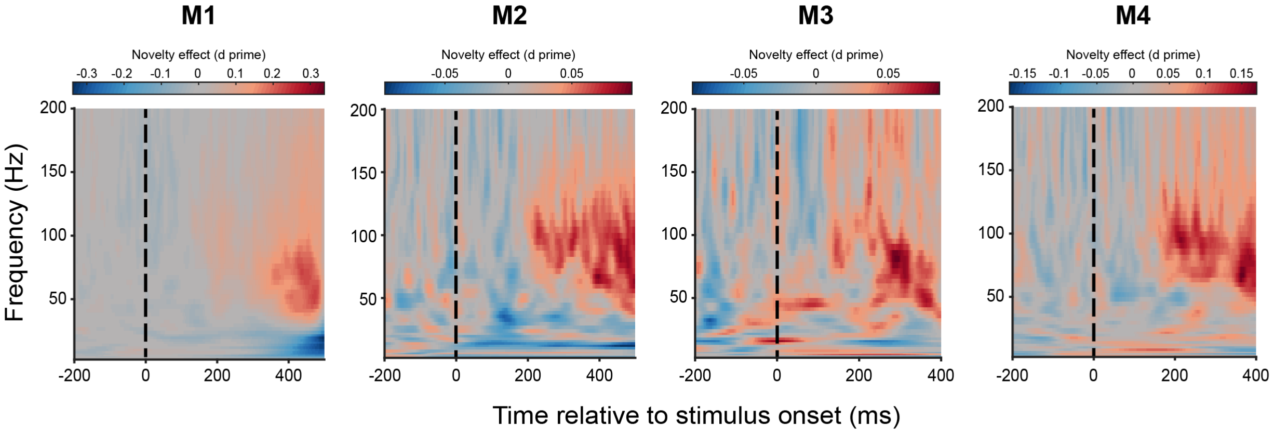

Figure S4. Repetition suppression evolves late and only at higher frequencies. Full spectrogram of d prime between the response to novel and repeated images. D prime was computed from the baseline-subtracted spectrogram of response to novel and repeated images for each channel and then averaged across channels.

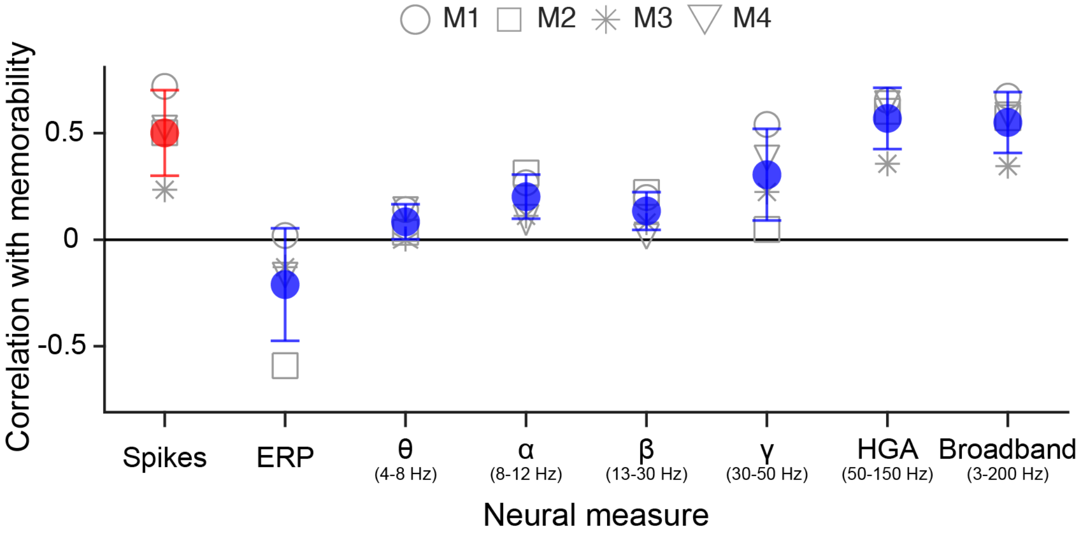

Figure S5. The correlation between memorability and spiking activity is only captured by high frequency components of the LFP (not low frequencies). Correlation between memorability score and response magnitude averaged over all channels for spikes (red) and several features of the LFP (blue). As was observed in novelty signals, the memorability correlation observed in spiking activity is only achieved in high frequencies of the field potential. Each point is a single monkey, colored points are the average across monkeys, and error bars are the standard deviation across all four monkeys.

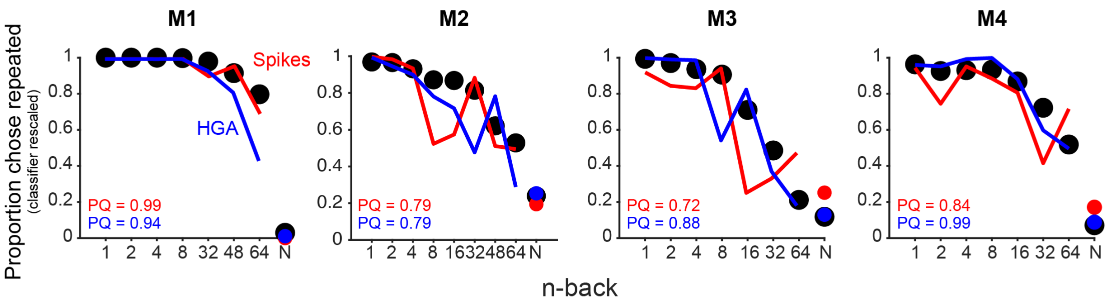

Figure S6. HGA predicts forgetting behavior as well as spikes. Neural predictions of the Fisher Linear Discriminant classifier trained on spikes (red) and HGA (blue) rescaled to match the level of behavioral performance (black) for all four monkeys (see Methods). Prediction Quality (PQ) values measure how well classifier performance predicts behavior (see Methods). The x-axis denotes the n-back for repeated images and the average performance for novel images is labeled as “N”.

bioRxiv preprint doi: https://doi.org/10.64898/2026.01.03.697516; this version posted January 4, 2026. The copyright holder for this preprint (which was not certified by peer review) is the author/funder, who has granted bioRxiv a license to display the preprint in perpetuity. It is made available under aCC-BY 4.0 International license.

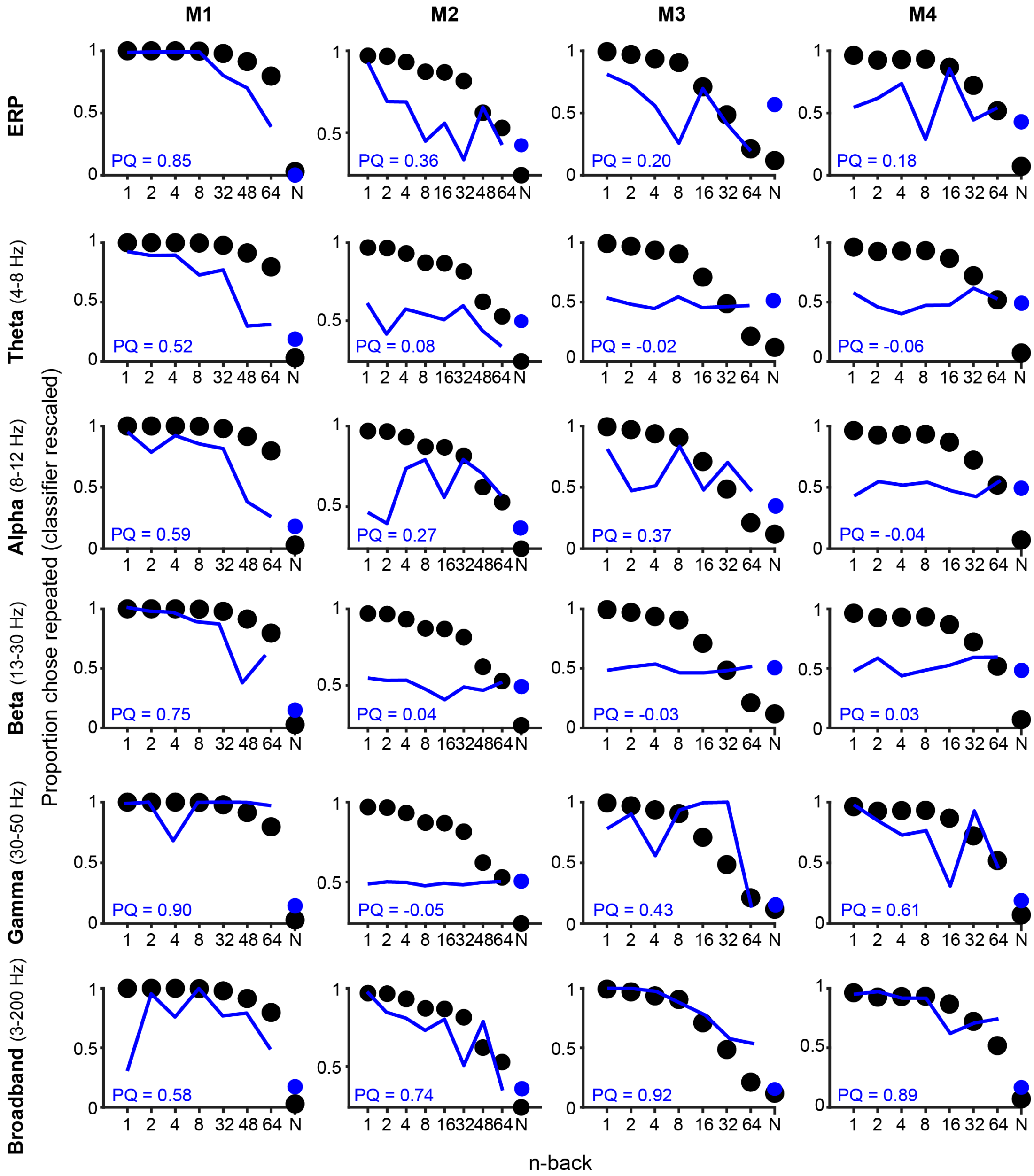

Figure S7. Other components of the LFP do not consistently accurately predict forgetting behavior. Neural predictions of the Fisher Linear Discriminant (see Methods) trained on other features of the LFP (blue) rescaled and plotted to match behavior (black) for all four subjects.

bioRxiv preprint doi: https://doi.org/10.64898/2026.01.03.697516; this version posted January 4, 2026. The copyright holder for this preprint (which was not certified by peer review) is the author/funder, who has granted bioRxiv a license to display the preprint in perpetuity. It is made available under aCC-BY 4.0 International license.

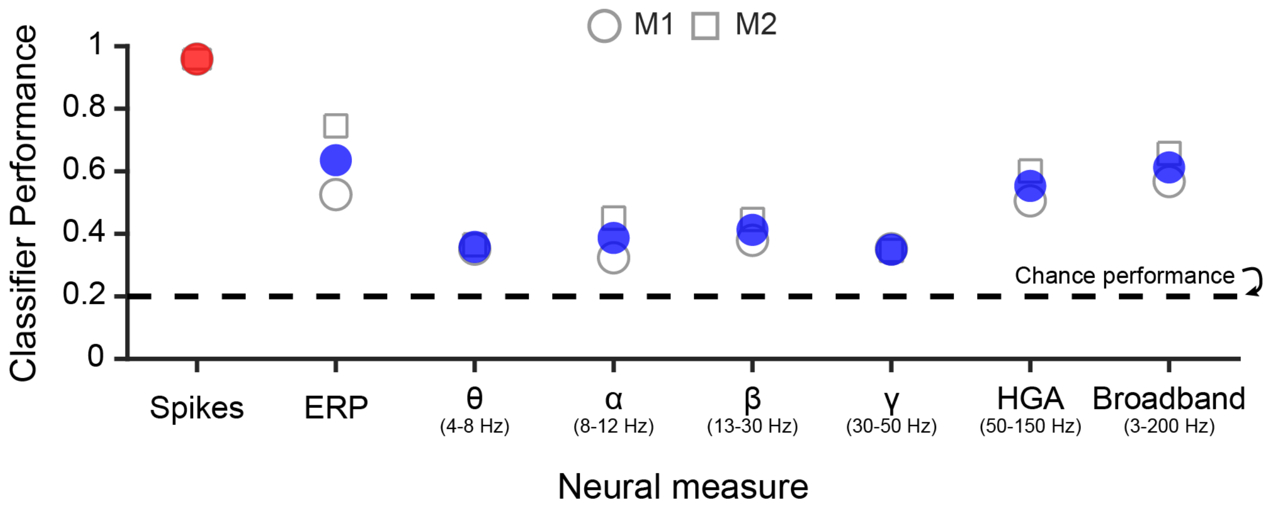

Figure S8. No components of the LFP show comparable categorical decoding performance to spiking activity. Prototype category decoder shown in Figure 5 with performance broken out as a function of frequency band. All frequency bands show worse decoding performance than spikes. Unlike the visual memory signals previously reported (novelty, recency, memorability), HGA is no closer to spikes than any other frequency band.

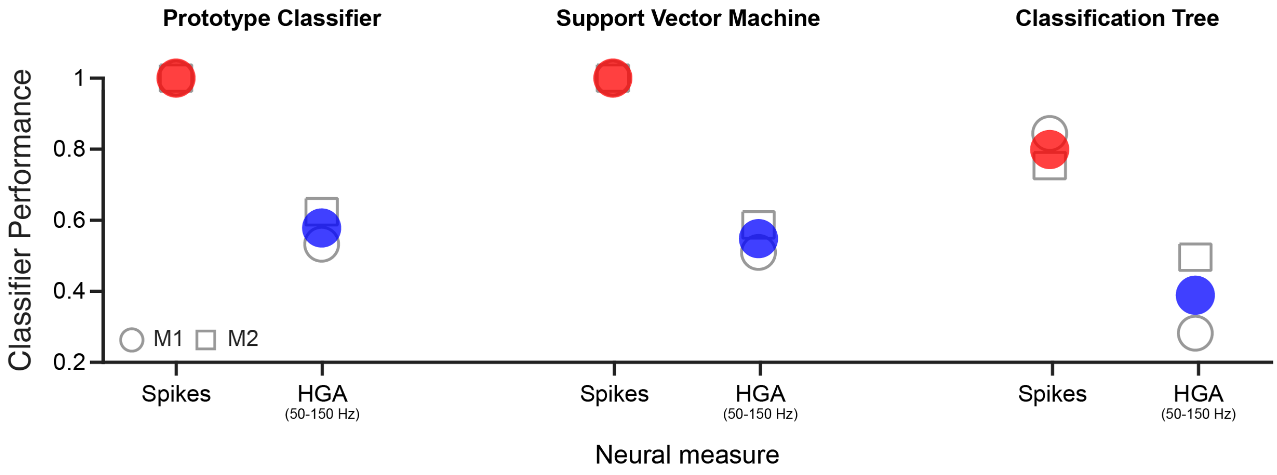

Figure S9. The claim that categorical representations are weaker in HGA than in spikes is not dependent on the type of classifier used. Performance of three types of categorical decoders in performing a 5-way image category classification: Prototype classifier (left, reported in Figure 5), support vector machine (middle), and classification tree (right). Chance is 0.2 for all decoders. Each shape represents classifier performance when trained on a single monkey and the colored circles are the mean across the two monkeys for spikes (red) and HGA (blue). For all three classifiers, performance is higher for spikes than HGA, indicating stronger categorical representations in spikes than HGA.

bioRxiv preprint doi: https://doi.org/10.64898/2026.01.03.697516; this version posted January 4, 2026. The copyright holder for this preprint (which was not certified by peer review) is the author/funder, who has granted bioRxiv a license to display the preprint in perpetuity. It is made available under aCC-BY 4.0 International license.

Supplementary Tables

Figure Panel	Statistical Test	M1	M2	M3	M4
1C	Pearson correlation	r(176)=-0.01, p=9.3e-1	r(176)=-0.23, p=6.5e-2	r(151)=-0.06, p=6.4e-1	r(151)=-0.47, p=2.5e-4
1E	Pearson correlation	r(66)=0.99, p=4.0e-59	r(66)=1.00, p=3.6e-65	r(56)=0.99, p=1.6e-45	r(56)=0.97, p=8.8e-42
2A	One-tailed Wilcoxon signed rank test	z=15.6, p=7.1e-55, r=0.93	z=9.6, p=3.8e-22, r=0.80	z=8.0, p=8.1e-16, r=0.81	z=12.8, p=6.0e-38, r=0.94
2B	One-tailed Wilcoxon signed rank test	z=18.2, p=4.0e-74, r=1.00	z=15.9, p=2.0e-57, r=1.00	z=10.5, p=3.5e-26, r=0.90	z=14.3, p=2.1e-46, r=0.99
2C Spikes v. HGA	Two sample t-test	t(878)=16.8, p=4.7e-55, d=1.13	t(686)=11.29, p=3.2e-27, d=0.86	t(452)=6.2, p=1.7e-9, d=0.58	t(566)=8.7, p=2.8e-17, d=0.73
3D	Pearson correlation	r(226)=0.72, p=1.3e-37	r(343)=0.50, p=2.0e-23	r(159)=0.23, p=2.7e-3	r(101)=0.55, p=2.1e-9
3E	Pearson correlation	r(226)=0.65, p=2.6e-29	r(343)=0.60, p=1.9e-35	r(159)=0.36, p=3.4e-6	r(101)=0.66, p=2.4e-14
3D v. 3E	Pearson correlation	r(226)=0.77, p=1.7e-46	r(343)=0.72, p=7.6e-57	r(159)=0.45, p=1.9e-9	r(101)=0.74, p=8.7e-19
5B Spikes	One sample t-test	t(694)=22.4, p=3.2e-84	t(663)=15.8, p=4.3e-48	-	-
5B HGA	One sample t test	t(694)=40.3, p=8.6e-184	t(663)=34.2, p=1.4e-148	-	-
5B Spikes v. HGA	Two sample t-test	t(1388)=11.3, p=1.5e-28	t(1326)=7.4, p=1.7e-13	-	-

Table S1. Statistical Tests. This table contains results of the statistical tests reported throughout the paper.
